# Uncertainty quantification for Bayesian active learning in rupture life prediction of ferritic steels

**DOI:** 10.1038/s41598-022-06051-8

**Published:** 2022-02-08

**Authors:** Osman Mamun, M. F. N. Taufique, Madison Wenzlick, Jeffrey Hawk, Ram Devanathan

**Affiliations:** 1 Energy and Environment Directorate, Pacific Northwest National Laboratory, Richland, USA; 2 Materials Performance Division, National Energy Technology Laboratory, 1450 Queen Avenue SW, Albany, OR 97321 USA; 3 Leidos Research Support Team, 1450 Queen Avenue SW, Albany, OR 97321 USA

**Keywords:** Metals and alloys, Computational methods

## Abstract

Three probabilistic methodologies are developed for predicting the long-term creep rupture life of 9–12 wt%Cr ferritic-martensitic steels using their chemical and processing parameters. The framework developed in this research strives to simultaneously make efficient inference along with associated risk, i.e., the uncertainty of estimation. The study highlights the limitations of applying probabilistic machine learning to model creep life and provides suggestions as to how this might be alleviated to make an efficient and accurate model with the evaluation of epistemic uncertainty of each prediction. Based on extensive experimentation, Gaussian Process Regression yielded more accurate inference ($Pearson\;correlation\;coefficent> 0.95$ for the holdout test set) in addition to meaningful uncertainty estimate (i.e., coverage ranges from 94 to 98% for the test set) as compared to quantile regression and natural gradient boosting algorithm. Furthermore, the possibility of an active learning framework to iteratively explore the material space intelligently was demonstrated by simulating the experimental data collection process. This framework can be subsequently deployed to improve model performance or to explore new alloy domains with minimal experimental effort.

## Introduction

Advanced ultra-supercritical power plants require increased steam temperature and pressure, for higher efficiency and lower carbon emissions, to comply with environmental regulations. Without building new power plants this can be achieved by increasing the operating temperature and pressures of the existing power plants. To design ferritic-martensitic steels (the most common cost-effective alloys used in power plants today) in the 9–12 *wt% Cr* range that can withstand these higher operating temperatures and pressures, a predictive model needs to be developed that can reliably and accurately predict the lifetime of an alloy and its interrelation to the factors that govern the mechanical and chemical properties of the alloys^1–3^. In this effort, machine learning has lent itself quite well to develop models with unprecedented accuracy with inference time orders of magnitude shorter than the traditional first-principles density functional theory^4–6^, Monte Carlo simulations^7^, molecular dynamics^8^, or phase field modeling^9–11^. Neural Network has been successfully employed in the past by Cole et al.^12^ and Brun et al.^13^ to model the creep rupture strength from physical and processing parameters. In a recent article, it was shown that a Gradient Boosting Algorithm^14^ can be used to efficiently predict the rupture life^15^ and rupture strength^16^ of 9–12 *wt% Cr* ferritic-martensitic steels and austenitic stainless-steels. However, oftentimes the prediction is unreliable for low confidence modeling, where the data is either scarce and highly non-linear or the uncertainty associated with the prediction is not available. Specifically, long-term creep rupture life exhibits uncertainty that ranges from several years to decades^17^. For proper reliability analysis, it is imperative to quantify the uncertainty associated with each data point, i.e., Epistemic uncertainty. It should be noted that there are two types of uncertainty associated with data sets: (1) Aleatoric uncertainty (i.e., resulting from the intrinsic nature of the data generation process), and (2) Epistemic uncertainty (i.e., resulting from the limitation of the model itself). In this article, three probabilistic machine learning approaches were used to make an efficient prediction with epistemic uncertainty estimate, namely; (1) Quantile Regression, (2) Natural Gradient Boosting, and (3) Gaussian Process Regression.

As a general rule the time span between the initial discovery of a novel material (i.e., the idea for a new alloy and the research to support further development) and integrating it into existing infrastructure can take more than 20 years^18^. By using artificial intelligence and machine learning, the screening process of identifying new candidate alloys that need to be synthesized and tested in a laboratory can be sped up, thereby greatly reducing the time span of the entire process. In this pursuit, the uncertainty estimate, from the probabilistic machine learning model, of the individual data points yet to be explored, can guide the candidate selection process via Bayesian Optimization in order to optimize for a certain desired property, e.g., longer creep rupture life or high yield stress. Another utility of the uncertainty estimate is to improve the existing machine learning model performance by acquiring data points that lead to maximum information gain (or greatest minimization of the entropy). Active learning has been demonstrated to be very effective in the design of experiments for the classification problem^19^; however, its application to the regression problem is still limited, owing to the sequential nature of the algorithm. In classification, only the decision boundary needs to be learned which implies using only a fraction of data points to build a reliable model. On the other hand, the regression problem requires learning over the whole data range, leading to a very slow data acquisition rate in a sequential learning fashion. In this research, a batch-mode, pool-based active learning framework has been demonstrated where data can be acquired in a parallel manner at each iteration by selecting candidates from several clusters (i.e., learned using unsupervised techniques).

## Probabilistic machine learning algorithms

### Quantile regression

Quantile regression forests are a non-parametric way of estimating the conditional quantiles of high dimensional predictor variables^20,21^. For this approach *Y* is defined as the response variable while *X* represents an array of predictor variables. In standard formalism, the regression analysis provides an estimate $\widehat{\mu }(x)$ of the conditional mean $(E(Y | X = x))$ for the response variable, which is obtained through *learning* the parameter of a regression model that minimizes the expected squared error loss:

1 $$E\left(Y \right| X=x)={\mathrm{argmin}}_{z}\;E\left\{{\left(Y-z\right)}^{2} \right| X=x\}$$

However, the conditional mean only captures the point estimate of the response variable. It does not provide any information about the distribution of the response variable at a certain point. The conditional distribution of $Y$ being smaller than $y$ given the predictor variable $X = x$ is given by,

2 $$F\left(y \right| X=x)=P\left(Y \le y \right|X=x)$$

The associated loss function to optimize this relation is given by the following equation,

3 $$\mathcal{L}= \left\{\begin{array}{l}\alpha \left|{y}_{i}-\widehat{{y}_{i}}\right| \quad if \left({y}_{i}-\widehat{{y}_{i}}\right)\ge 0\\ \left(\alpha -1\right)\left|{y}_{i}-\widehat{{y}_{i}}\right| \quad if \left({y}_{i}-\widehat{{y}_{i}}\right)< 0\end{array}\right.$$

here, $\alpha$ ranges from 0 to 1 depending on the percentile that one wishes to achieve. To get the $95\%$ prediction interval, two models are fit with $\alpha =0.025$ and $0.975$. In this work, in addition to the prediction interval, the median response variable is also computed using $\alpha =0.5$. For quantile regression, Gradient Boosting Decision Tree (GBDT) is used which was the best performing non-probabilistic model in a previous study^15^. GBDT is an ensemble of weak decision tree models that iteratively fit data to minimize the error made in the previous iteration^14^.

### Natural gradient boosting regression

Natural Gradient Boosting (NG Boosting) algorithm is another probabilistic GBDT based model; however, unlike quantile regression, or conditional mean estimation, it learns the full probability distribution by construction^22^. The key idea in NG Boosting is to use the natural gradients instead of the regular gradients, thus allowing the algorithm to fit a probability distribution over the outcome space, conditioned on the predictor variables. The algorithm consists of three distinct components: (1) Base learner, (2) Parametric distribution, and (3) Scoring rule. The base learner used in the algorithm is the GBDT. Instead of making a point estimate, a probability distribution *learns* by learning the parameter of the distribution, e.g., the mean and standard deviation in the case of Gaussian distribution. The scoring rule is selected in such a way that the forecasted probability distribution gets a high score if it matches the true distribution with respect to the observation $y$. In the algorithm, negative log-likelihood is used as the scoring rule.

### Gaussian process regression

Gaussian Process Regression (GPR) is a flexible class of non-parametric models^23^, where the non-linearity in the data can be modeled by incorporating different basis functions within the kernel to compute the covariance matrix. It can also include user-defined prior functions to take advantage of domain knowledge (which is crucial when data are scarce). The GPR prior is an infinite-dimensional, multi-variate distribution that can be completely described by a mean function $\left(m\left(x\right)\right)$ and a covariance function $k(x, x{^{\prime}})$,

4 $$f\left(x\right) \sim \mathcal{G}\mathcal{P}(m\left(x\right), k(x, x{^{\prime}}))$$

The posterior predictive distribution is then obtained by computing the likelihood from the observed data within a Monte Carlo framework. However, it can also be posed as an optimization problem to minimize the analytical negative log-likelihood. Once the learning process is complete, the posterior distribution not only provides a point estimate for the prediction but also a standard deviation of the prediction.

### Active learning

**Figure 1:**
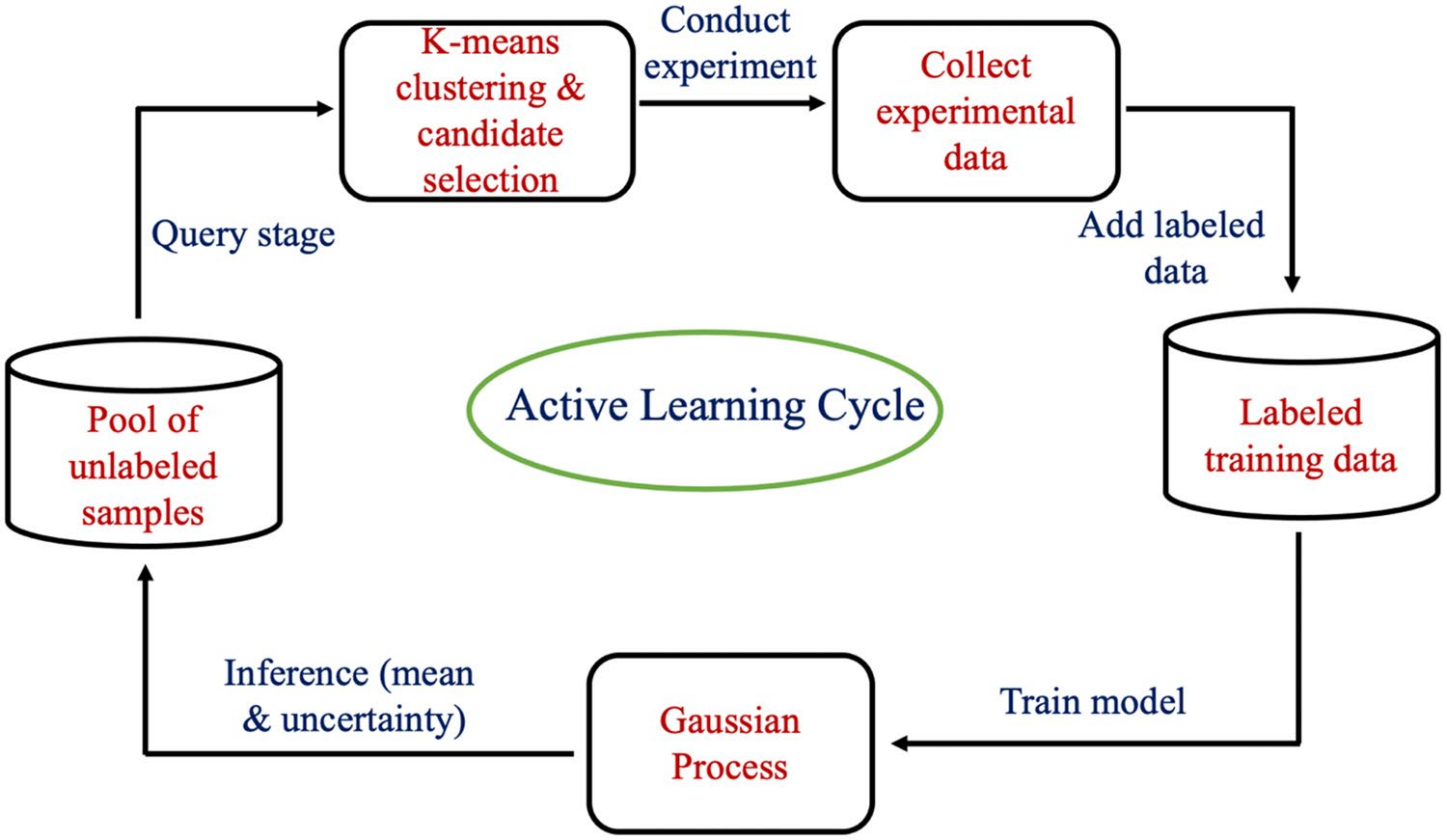
Active learning workflow of a batch-mode, pool-based method. In the query stage, candidates are selected from the pool of unlabled samples based on K-means algorithms and variance reduction approach. The selected samples are tested in the laboratory and added to the training data to make refined inference on the pool of unlabeled samples.

Active learning is a machine learning method that is used for optimal design of experiments by iteratively guiding the selection of the next unexplored data points to be acquired by using a suitable acquisition function^19^. These selected new data, when added to the already explored training data, will yield the maximum improvement in the model performance thus leading to a better model with fewer data points acquired. In this work, a pool-based Bayesian active learning method based on GPR as the base learner is used which selects the most useful samples from a pool of unlabeled samples. However, the traditional pool-based sequential active learning is not suitable for an iterative exploration of 9–12 *wt% Cr* ferritic-martensitic steel space, as each experiment is expensive and can take several days to years to complete. Instead of choosing one sample at each iteration, a batch mode is adapted which enables selecting multiple samples at each iteration. In Fig. 1, a schematic of the active learning loop is shown to illustrate the inner working of the active learning cycle adapted in this work. The algorithm used for active learning for batch mode in this study is the same as the standard approach; except, during the query stage, the unpooled data are clustered using k-means algorithm. At this point the variance reduction approach (i.e., selecting the most uncertain sample) is used to select one sample from each cluster. This approach is not only faster than the traditional sequential active learning, but it is also better in terms of *informativeness* (which represents the ability of the sample to reduce the generalization error in the adopted model and ensures lower uncertainty in the subsequent iteration), and diversity in the samples (meaning the data is the most dissimilar to the data already present in the training set). Since the data are clustered into several groups, each selected sample is diverse from another, and the variance reduction approach ensures that the samples are also very informative.

## Methods

**Figure 2:**
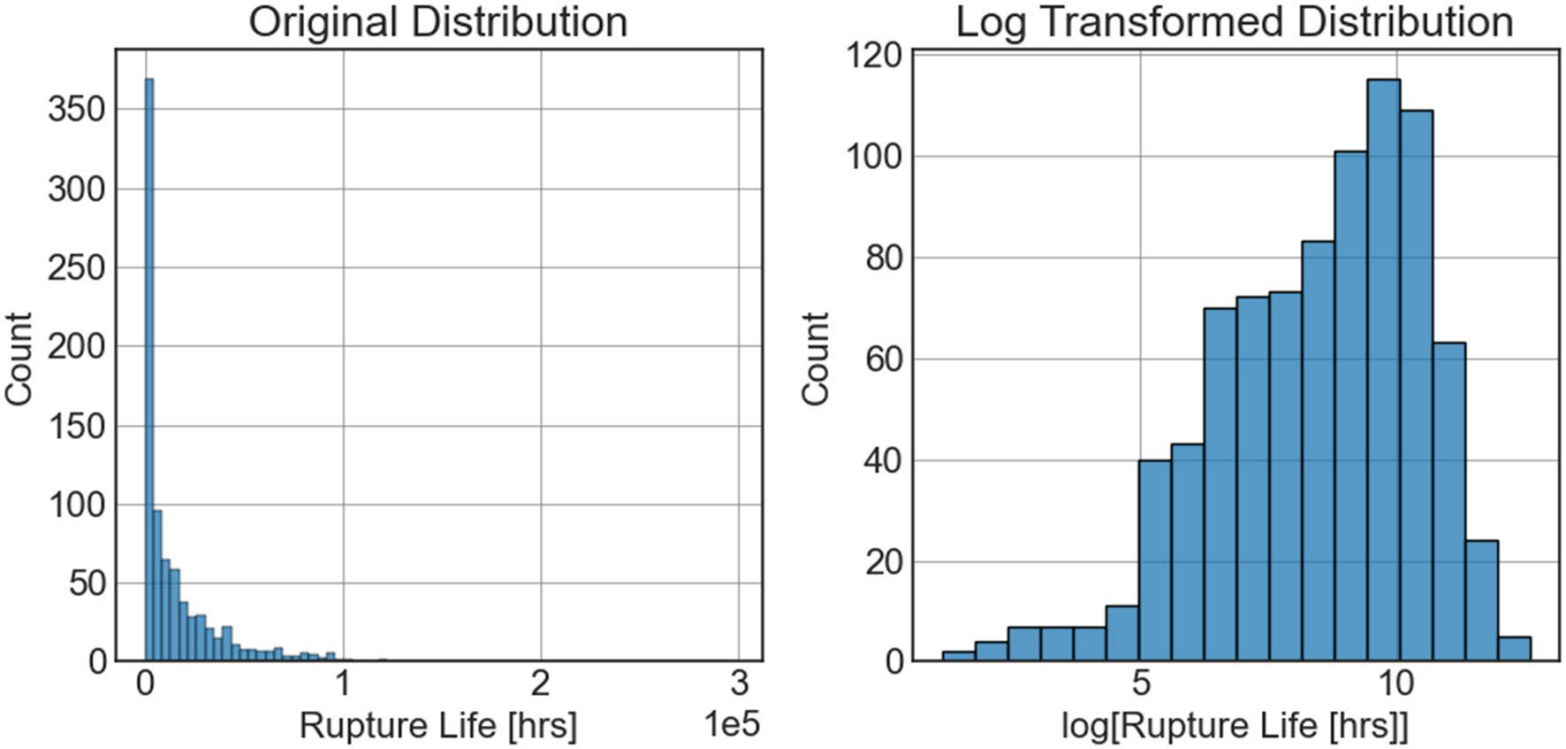
The distribution of rupture life in both original and transformed space.

The dataset for 9–12 *wt% Cr* ferritic-martensitic steel was collected and compiled by the National Energy Technology Laboratory (NETL) as part of the Extreme Environment Materials (eXtremeMAT or XMAT), a DOE-funded project that seeks to reduce the time to bring new alloys to commercial readiness while at the same time providing the modeling framework necessary to describe its entire life in operation at all length scales. In Table 1, we show the mean, median, maximum and minimum value of each features used in the machine learning model. The dataset contains 875 rows of data corresponding to each unique composition and processing parameter set. The preprocessing step consists of imputation of missing data by mean values, normalization of the data using standard scaler approach. The creep rupture life, or the target variable, is always positive, resulting in inherent constraints that need to be incorporated into the machine learning algorithms. It is a particularly severe problem for probabilistic modeling as the response variable is allowed to have any value with certain probabilities, albeit very small for values outside the 95% confidence interval. Due to this freedom in probabilistic modeling, it is crucial to log-transform the data so that the predicted response variable is always positive. In Fig. 2, the distribution of response variables in their original space and their transformed space is shown. This transformation also makes the data more normally distributed which helps tackle the heteroscedasticity problem to some extent.

**Table 1:** Summary of the features present in 9–12% Cr dataset, including feature name, description, units, mean values, standard deviations, maximum, and minimum values.

Feature name	Description	Unit	9–12% Cr FMA			
			Mean	Std	Maximum	Minimum
$Fe$	Iron content	$wt\%$	$86.70$	$2.40$	$89.59$	$78.61$
$C$	Carbon content	$wt\%$	$0.12$	$0.04$	$0.25$	$0.05$
$Cr$	Chromium content	$wt\%$	$10.02$	$1.48$	$12.90$	$8.31$
$Mn$	Manganese content	$wt\%$	$0.51$	$0.11$	$0.98$	$0.05$
$Si$	Silicon content	$wt\%$	$0.31$	$0.06$	$0.86$	$0.01$
$Ni$	Nickel content	$wt\%$	$0.28$	$0.22$	$0.90$	$0.04$
$Co$	Cobalt content	$wt\%$	$0.60$	$1.05$	$8.22$	$0.01$
$Mo$	Molybdenum content	$wt\%$	$0.65$	$0.33$	$2.03$	$0.01$
$W$	Tungsten content	$wt\%$	$0.82$	$0.84$	$2.91$	$0.01$
$Nb$	Niobium content	$wt\%$	$0.05$	$0.03$	$0.09$	$0.01$
$Al$	Aluminum content	$wt\%$	$0.01$	$8.69 \times {10}^{-3}$	$0.04$	$1.00\times {10}^{-3}$
$P$	Phosphorous content	$wt\%$	$0.01$	$6.65 \times {10}^{-3}$	$0.02$	$1.00{\times 10}^{-3}$
$Cu$	Copper content	$wt\%$	$0.23$	$0.35$	$0.97$	$2.00 \times {10}^{-3}$
$Ti$	Titanium content	$wt\%$	$9.00 \times {10}^{-3}$	$0.02$	$0.10$	$1.00 \times {10}^{-3}$
$V$	Vanadium content	$wt\%$	$0.18$	$0.07$	$0.30$	$0.01$
$B$	Boron content	$wt\%$	$4.00 \times {10}^{-3}$	$3.00 \times {10}^{-3}$	$0.01$	$7.00 \times {10}^{-4}$
$N$	Nitrogen content	$wt\%$	$0.04$	$0.02$	$0.07$	$0.01$
$S$	Sulfur content	$wt\%$	$3.36 \times {10}^{-3}$	$3.47 \times {10}^{-3}$	$1.70{\times 10}^{-3}$	$3.00 {\times 10}^{-4}$
$Zr$	Zirconium content	$wt\%$	$1.00 \times {10}^{-3}$	$3.37 \times {10}^{-6}$	$1.00{\times 10}^{-3}$	$1.00{\times 10}^{-3}$
$Normal$	Normalization or Austenization heat treatment temperature	$^\circ{\rm C}$	$1044.46$	$29.48$	$1150.00$	$900.00$
$Temper1$	Temper heat treatment 1	$^\circ{\rm C}$	$744.58$	$56.18$	$860.00$	$570.00$
$AGS No.$	Austenitic grain size number	$\frac{grains}{{mm}^{2}}$	$7.73$	$2.52$	$10.70$	$0.50$
$CT\_Temp$	Creep test temperature	$^\circ{\rm C}$	$589$	$72$	$750$	$450$
$CT\_EL$	Elongation to failure	%	$32.65$	$21.7$	$146.00$	$0.70$
$CT\_RA$	Reduction in area	%	$69.50$	$24.66$	$98$	$0$
$log\_CT\_CS$	Logarithm of creep test stress		$4.93$	$0.065$	$6.25$	$2.30$
$log\_CT\_MCR$	Logarithm of minimum creep rate		$-7.56$	$2.49$	0.21	– 8.97

For Quantile regression and NG Boosting, the predicted response variable was computed by taking the exponential of the prediction for all the quantiles. However, for GPR the mean and standard deviation of the log-transformed prediction of individual points must be transformed with the following formula to achieve the mean and standard deviation in the original space.

5 $${\mu }_{y}={e}^{({\mu }_{\mathrm{log}\left(y\right)}+ \frac{{{\sigma }_{\mathrm{log}(y)}}^{2}}{2})}$$

6 $${\sigma }_{y}=\sqrt{{\mu }_{y}^{2}{ \times \sigma }_{\mathrm{log}(y)}^{2}}$$

where ${\mu }_{\mathrm{log}\left(y\right)}$ and ${\sigma }_{\mathrm{log}\left(y\right)}$ are mean and standared deviation of the prediction in the log-transformed sapce, and ${\mu }_{y}$ and ${\sigma }_{y}$ are the mean and standard deviation of the prediction in the original space, respectively. For Quantile Regression, the CatBoost^24^ python package was used with scikit-learn API. For NG Boosting, the NGBoost^22^ python package was used. To perform GPR, scikit-learn^25^ python package was used. The code to reproduce the machine learning models and active learning results can be obtained from https://github.com/mamunm/uncertainty_quantification_creep_life.

To quantify the overall model performance for prediction, a five-fold, cross-validation scheme was used where in each iteration 80% data were used to train the model and 20% data were used to test the model performance. Pearson correlation coefficient (PCC) was used to measure the predictive performance and coverage (i.e., the fraction of data that lie within two (2) standard deviations) was used to quantify the reliability of the uncertainty estimate.

For active learning, $20\%$ data were used as test data and then 20% of the remaining data were used as the initial training data. Kmeans clustering algorithm is used to fit the remaining data into 10 clusters. In each iteration, 10 new data points are added to the training data by selecting 1 point each from the 10 clusters. The correlation coefficient of the holdout test set is then calculated to determine the accuracy of the model at each iteration. The active learning technique is compared to a baseline random sample addition to indicate the relative efficiency of the active learning model.

### Disclaimer

This manuscript was prepared as an account of work sponsored by an agency of the United States Government. Neither the United States Government nor any agency thereof, nor any of their employees, makes any warranty, express or implied, or assumes any legal liability or responsibility for the accuracy, completeness, or usefulness of any information, apparatus, product, or process disclosed, or represents that its use would not infringe privately owned rights. Reference therein to any specific commercial product, process, or service by trade name, trademark, manufacturer, or otherwise does not necessarily constitute or imply its endorsement, recommendation, or favoring by the United States Government or any agency thereof. The views and opinions of authors expressed therein do not necessarily state or reflect those of the United States Government or any agency thereof.

## Results and discussion

**Figure 3:**
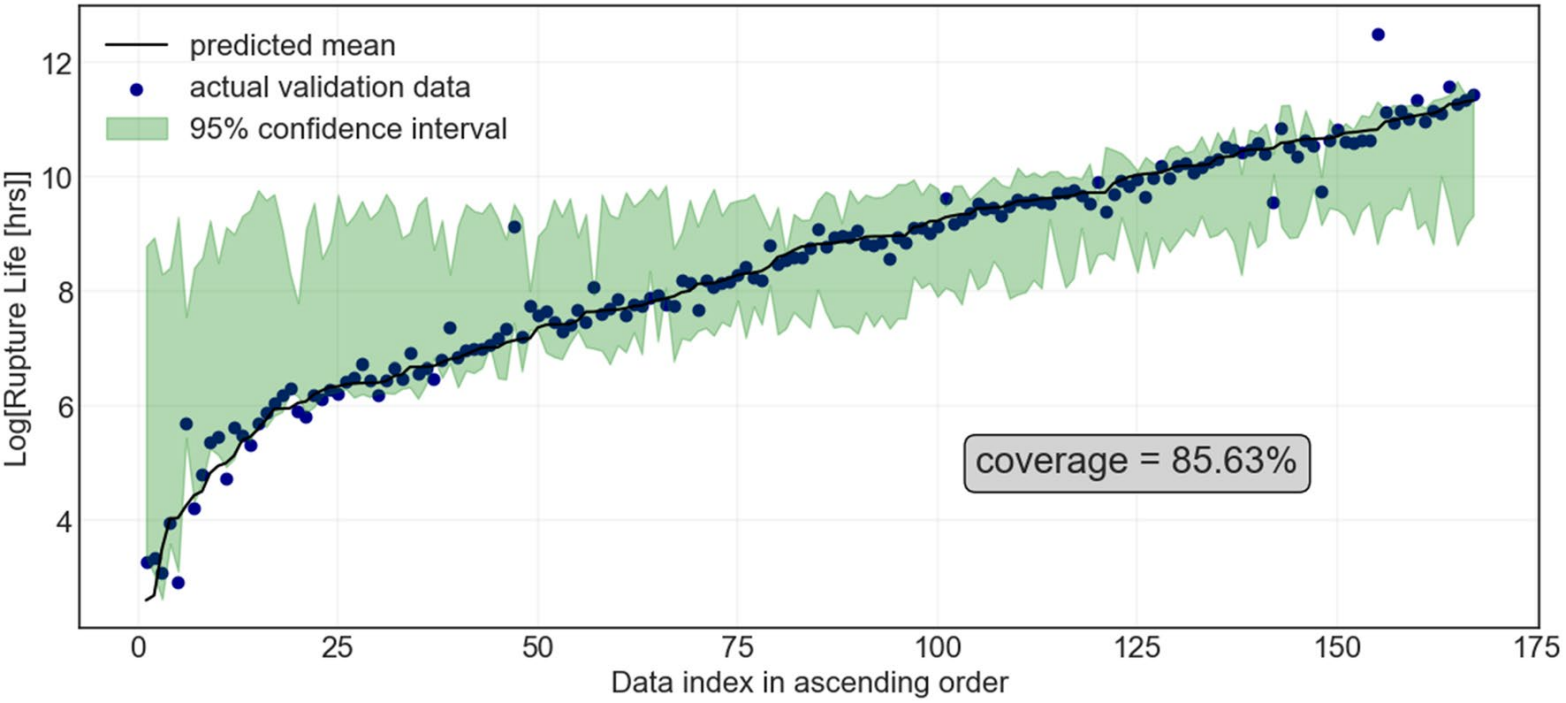
Actual and Quantile Regression predicted creep rupture life (in log scale) of ferritic-martensitic steels in ascending order of the predicted creep rupture life. The green area indicates the 95% prediction interval.

Figure 3 illustrates the actual rupture life and the predicted rupture life with the $95\%$ confidence interval for prediction on the hold-out test set. PCC for training and testing data is 0.$980 \pm 0.006$ and 0.$890\pm 0.050$, respectively, with the coverage ranging from $95.34 \pm 0.77 \%$ and $80.74 \pm 3.10 \%$, respectively. The accuracy of the model is quite satisfactory as discussed in our previous work^15^. In addition to that, the model can now provide a 95% prediction interval that contains about 80% of the test dataset. There are several problems associated with quantile regression that questions the reliability of the model for real-world applications.

The first and obvious problem is the lower prediction interval $(|{y}_{median}-{y}_{lower}|)$ is greater than the higher prediction interval $(|{y}_{higher}-{y}_{median}|)$ in the high rupture life area and vice versa in the low rupture life area. Due to the inherent nature of the loss function of the quantile regression, it overestimates the lower prediction interval for this dataset as there are significantly more data in that region. Another problem is that in a few instances the predicted median is not within the prediction interval. Since three models are being optimized for median and two (2) standard deviations in the interquartile range, each model optimizes the parameters irrespective of the other models. As a result, inconsistency appears in the prediction interval and predicted median. The third and final problem is the confidence interval is not well-calibrated which questions the reliability of the uncertainty estimate. For a well-calibrated model, 95% of the data should fall within two (2) standard deviations which is satisfied for the training data but only $80.74 \pm 3.10 \%$ of the testing data are contained within two standard deviation leading to under-estimation of the confidence interval.

**Figure 4:**
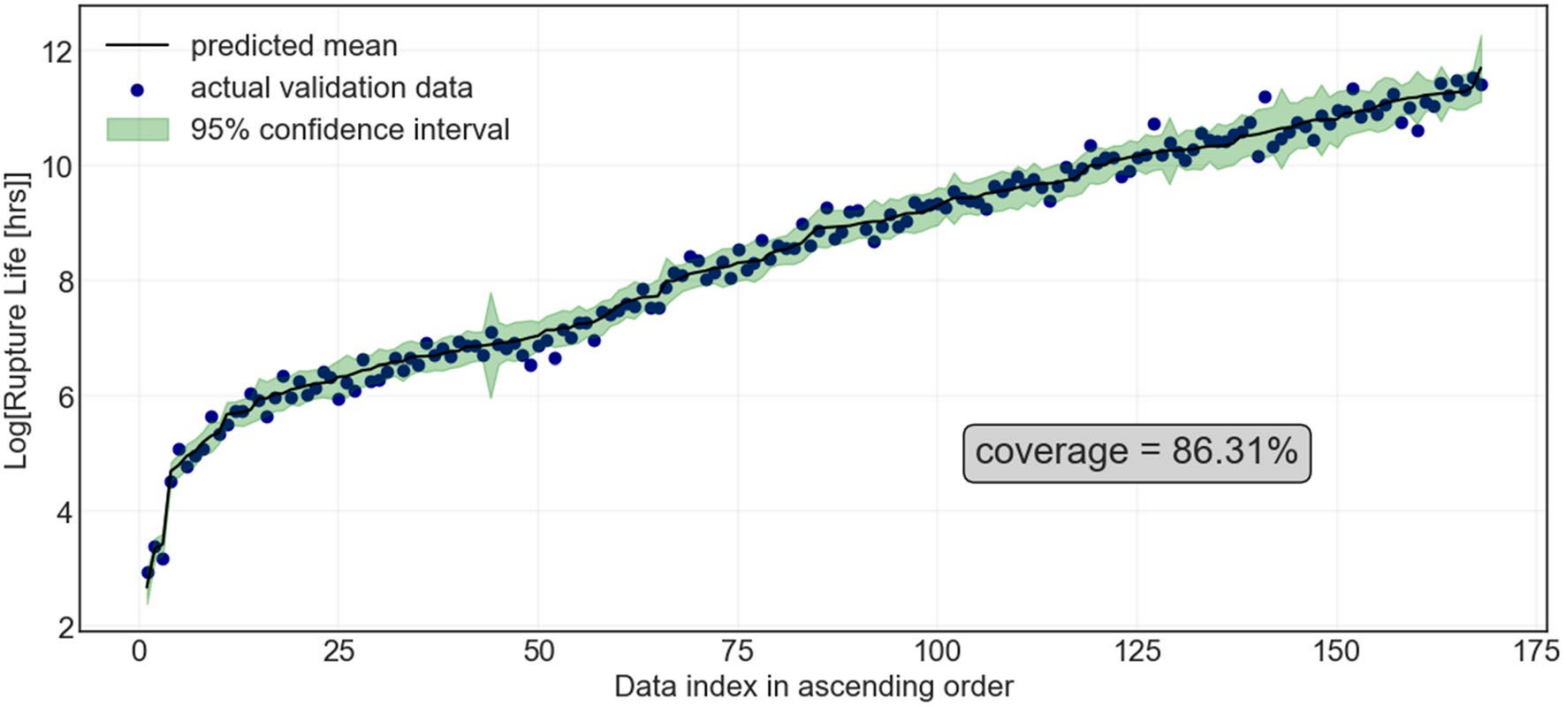
Actual and NG Boosting predicted creep rupture life (in log scale) of ferritic-martensitic steels in ascending order of the predicted creep rupture life. The green area indicates the 95% prediction interval.

In Fig. 4, the actual and NG Boosting predicted rupture life along with the prediction interval is shown for the hold-out test set. The PCC for the training and testing set is $0.980 \pm 0.002$ and $0.920\pm 0.030$, respectively, with the coverage ranging from $98.47 \pm 0.28 \%$ and $84.08 \pm 2.03 \%$, respectively. Surprisingly, the model prediction is more accurate than the non-probabilistic Gradient Boosting approach. Furthermore, the uncertainty estimate is better behaved than the quantile regression. The prediction interval always contains the mean and the uncertainty is higher where the data is scarce, leading to a more faithful model that can be used for an iterative exploration of the materials space. However, the uncertainty is not well-calibrated as evident by this coverage of the testing data, i.e., $84.08 \pm 2.03 \%$.

**Figure 5:**
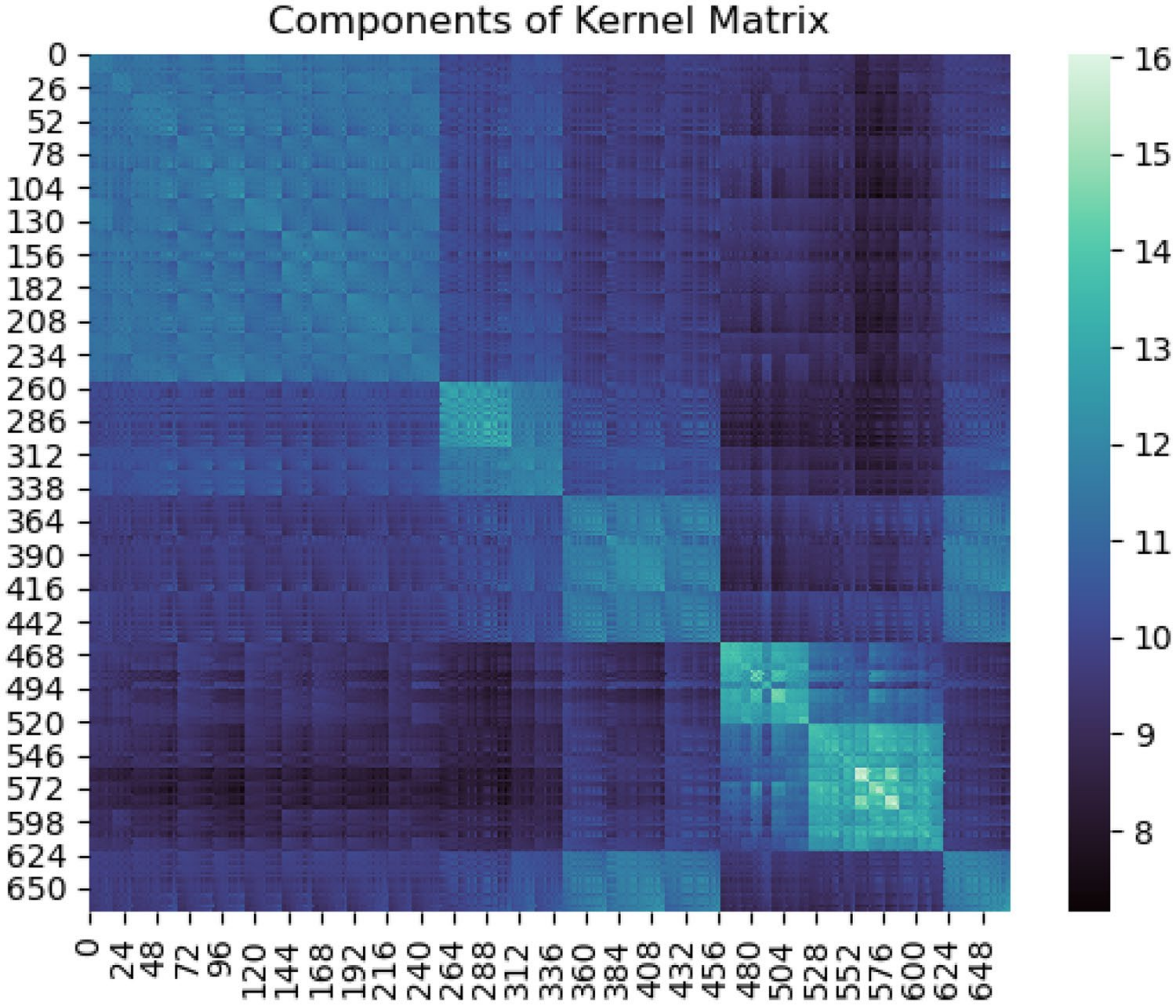
Components of kernel matrix for the additive kernel used in this study. The x and y axes represent the index of the samples and the color bar shows the magnitude of the kernel function.

Next, the GPR model was trained with an additive kernel consisting of a Matern kernel, a white kernel, and a dot product kernel. The Matern kernel models the short-range interaction present in the data. The white kernel adds noise to the diagonal elements of the kernel matrix to make it more robust and generalizable to noise. The dot product kernel models any linear trends between the data points. In Fig. 5, the kernel matrix is illustrated for the training data. As expected, there is a high correlation between adjacent data points but the correlation slowly fades with increasing distance. Also, the data seem to be clustered naturally into about six (6) distinct clusters.

**Figure 6:**
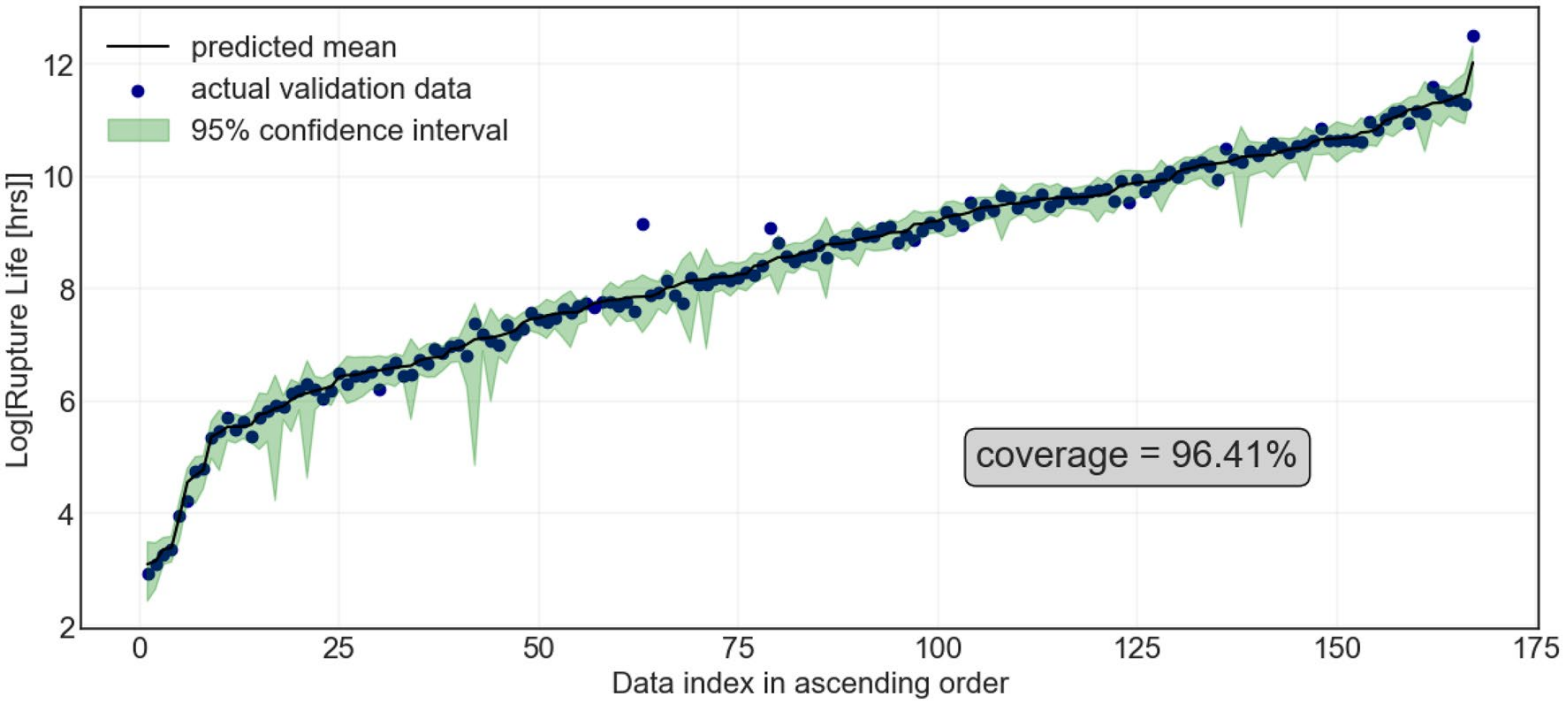
Actual and Gaussian Process predicted creep rupture life (in log scale) of ferritic-martensitic steels in ascending order of the predicted creep rupture life. The green area indicates the 95% prediction interval.

Next, Fig. 6 illustrates the actual and predicted creep rupture life for the GPR along with the $95\%$ prediction interval. The PCC for training and testing was set at $0.990 \pm 0.001$ and $0.970\pm 0.020$, respectively, with the coverage ranging from $99.25 \pm 0.18 \%$ and $96.40 \pm 1.52 \%$, respectively. Not only is the GPR highly accurate compared to the other two algorithms, but it also has very high coverage which means more than 96% of the data lies within the 95% prediction interval, as it should be for a well-calibrated uncertainty estimation.

### Active learning for iterative exploration

**Figure 7:**
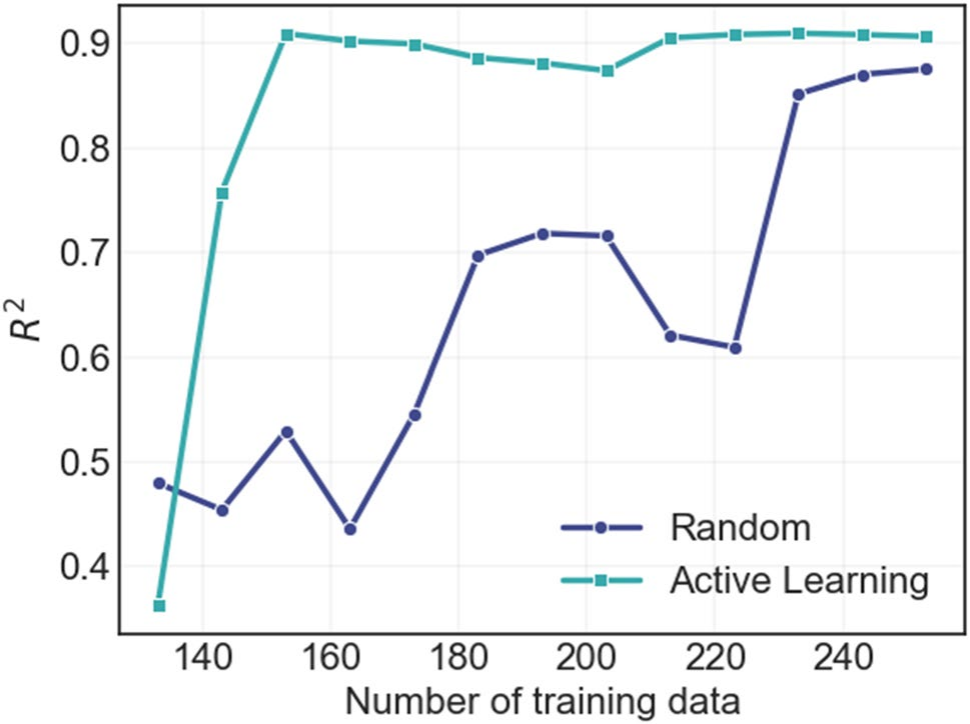
Correlation coefficient of the hold-out test set for both random data acquisiton and active learning algorithm.

The data used in this study were collected over 30 years of concerted efforts from government and non-government initiatives^16^. The overarching goal of probabilistic machine learning is to make reliable predictions for unknown alloys as well as to accelerate the data collection process to improve the model performance. This process exhaustively explores a material’s space with minimal experimental effort. To this end, the uncertainty of prediction provides valuable information about the knowledge gap present in the dataset, and by acquiring the data with the highest uncertainty, significant improvement in the model’s performance can be achieved. In Fig. 7, the correlation coefficient for the hold-out test data for both the random and the active data collection processes are shown. Not surprisingly, the active learning process can readily find the data points that lead to the most improvement in test set performance. This technique therefore can be used to design the experimentation necessary both to improve the model performance and to optimize the desired alloy properties. By incorporating this framework into the experimental/computational data collection process, significant improvement in materials discovery can be achieved.

## Conclusion

In this work, three approaches are described to determine the uncertainty associated with machine learning prediction of creep rupture life of ferritic-martensitic steels for each data point. The advantages and disadvantages of each approach was described with the accuracy of the prediction based on coverage of the 95% prediction interval. From this effort, the GPR was identified as the best algorithm for both accurate inference and uncertainty estimation. Implementing this uncertainty estimation technique in the machine learning workflow will enable researchers to estimate the variance in the predicted values and to understand the risk inherent in the model. This technique will further help to ensure model interpretability by providing a 95% confidence interval for the predicted values. Finally, a simulated design of experiments was used to iteratively collect data using an active learning framework to accelerate data collection for reliable and informative data acquisition. Implementing this technique will enable researchers to more efficiently explore the alloy design space, and optimize the experimentally collected data points for improving the model predictions. These methods will ultimately help optimize the desired properties of the alloys.

## Data Availability

The data used that support the findings of this study are available upon request to M. W. (Madison.Wenzlick@NETL.DOE.GOV).
